# Endocrine Dysfunctions in Patients with Inherited Metabolic Diseases

**DOI:** 10.4274/jcrpe.2288

**Published:** 2016-09-01

**Authors:** Şahin Erdöl, Halil Sağlam

**Affiliations:** 1 Uludağ University Faculty of Medicine, Department of Pediatrics, Division of Metabolism, Bursa, Turkey; 2 Uludağ University Faculty of Medicine, Department of Pediatrics, Division of Pediatric Metabolism and Endocrinology, Bursa, Turkey

**Keywords:** Inherited, metabolic diseases, endocrine dysfunction, children

## Abstract

**Objective:**

Inherited metabolic diseases (IMDs) can affect many organ systems, including the endocrine system. There are limited data regarding endocrine dysfunctions related to IMDs in adults, however, no data exist in pediatric patients with IMDs. The aim of this study was to investigate endocrine dysfunctions in patients with IMDs by assessing their demographic, clinical, and laboratory data.

**Methods:**

Data were obtained retrospectively from the medical reports of patients with IMDs who were followed by the division of pediatric metabolism and nutrition between June 2011 and November 2013.

**Results:**

In total, 260 patients [139 males (53%) and 121 females (47%)] with an IMD diagnosis were included in the study. The mean age of the patients was 5.94 (range; 0.08 to 49) years and 95.8% (249 of 260 patients) were in the pediatric age group. Growth status was evaluated in 258 patients and of them, 27 (10.5%) had growth failure, all cases of which were attributed to non-endocrine reasons. There was a significant correlation between growth failure and serum albumin levels below 3.5 g/dL (p=0.002). Only three of 260 (1.1%) patients had endocrine dysfunction. Of these, one with lecithin-cholesterol acyltransferase deficiency and another with Kearns-Sayre syndrome had diabetes, and one with glycerol kinase deficiency had glucocorticoid deficiency.

**Conclusion:**

Endocrine dysfunction in patients with IMDs is relatively rare. For this reason, there is no need to conduct routine endocrine evaluations in most patients with IMDs unless a careful and detailed history and a physical examination point to an endocrine dysfunction.

**WHAT IS ALREADY KNOWN ON THIS TOPIC?**

Inherited metabolic diseases (IMDs) can affect many organ systems, including the endocrine system.

**WHAT THIS STUDY ADDS?**

There is limited data regarding endocrine dysfunctions related to IMDs in adults, but no data exist in pediatric patients with IMDs.

## INTRODUCTION

The clinical disorders that arise from a single gene defect and develop as a consequence of a blockage of the metabolic pathways are accepted as inherited metabolic diseases (IMDs). Metabolic disorders fall into three distinct groups depending on their occurrence mechanism-intoxication type, energy deficit type, and disorders affecting the degradation of complex molecules (1).

Patients with a metabolic disease may have serious endocrine problems. Many endocrine glands are affected; however, diabetes mellitus (DM), thyroid dysfunction, and gonadal deficiency are more frequent (2). Endocrine disorders, especially DM, also tend to be more common in patients with mitochondrial diseases (3). It is notable that many latent endocrine dysfunctions may be concomitant with Fabry disease and could be life threatening (4). As IMDs may be accompanied by serious endocrine dysfunctions, being aware of the condition and early diagnosis by simple tests are crucial to preventing possible complications (2).

In this study, we aimed to perform an endocrine assessment in patients diagnosed with IMDs.

## METHODS

Ethical approval from the local ethics committee was obtained before the study. The data were collected from the records of 260 patients with IMDs followed in our unit between June 2011 and November 2013. The ages of the patients ranged from 0.08 to 49 years.

Demographic findings (i.e., age, gender), results of anthropometric measurements (i.e., height, weight, head circumference), and physical examination outcomes were evaluated. Annual growth rate and bone age of the patients were calculated. Patients with a height standard deviation score below -2 were accepted as having growth retardation. In patients with growth retardation, growth hormone (GH) deficiency was assessed by growth velocity, bone age, serum insulin-like growth factor 1 (IGF-1) value, and GH stimulation tests when needed.

Serum glucose and hemoglobin A1c levels of the patients were evaluated for DM. Serum sodium (Na) and potassium (K) levels were measured to assess mineralocorticoid functions of the adrenal gland. Adrenocorticotropic hormone (ACTH) and cortisol levels were used for the evaluation of glucocorticoid functions of the adrenal cortex. Serum thyroid-stimulating hormone (TSH) and free thyroxine (fT^4^) levels were assessed for the evaluation of thyroid function. Serum calcium (Ca), phosphorus (P), and alkaline phosphatase (ALP) levels were used to evaluate bone metabolism. In addition, serum albumin levels were measured to assess the nutrition status of the patients.

For statistical analysis, SPSS for Windows version 16.0 software was employed. Shapiro-Wilk test was used to determine whether data distribution was normal. Arithmetic means ± standard deviations (SD) were calculated. Difference between categorical variables (frequencies) was assessed by the chi-square test. For continuous variables, means of two groups were compared with student’s t-test. Correlation between variables was calculated using Pearson’s coefficient. For significance, a=0.05 (p<0.05) was used.

## RESULTS

Of 260 patients with IMD, 139 (53%) were males and 121 (47%) were females. The numbers of the patients included in each of the main and sub-groups of disease are shown in Table 1. No significant differences regarding gender within the main (p=0.145) group and sub-groups (p=0.232) of disease were found. A total of 249 (95.8%) patients were in the pediatric age group and 11 (4.22%) were in the adult age group.

Growth retardation was observed in 27 of 258 patients (10.5%). Eight patients (29.6%) were in the cellular intoxication group, 11 (40.7%) were in the energy deficit group, and 8 (29.6%) were in the complex molecule accumulation-induced main disease group (Table 1). None of the patients with growth retardation had an abnormal GH/IGF-1 axis.

The proportion of patients who were on dietary protein restriction for their primary diagnosis of IMD was 21.5%. There was no correlation between protein restriction and presence of growth retardation (p=0.712); however, a significant correlation (p=0.002) was found between a serum albumin level below 3.5 g/dL and growth retardation. In addition, growth retardation was observed in 6 of 14 patients (42.9%) with a serum albumin level below 3.5 g/dL.

Only 2 (0.07%) patients with IMD had DM. The primary diagnosis of one of these two patients was lecithin-cholesterol acyltransferase (LCAT) deficiency and that of the other patient was Kearns-Sayre syndrome (KSS). Glucocorticoid deficiency was observed only in one (0.4%) patient with glycerol kinase deficiency. None of the patients with IMD had pubertal or GH disorders nor disorders related to thyroid, mineralocorticoid hormones, or bone disorders.

## DISCUSSION

In previous studies, mainly conducted on adult patients, endocrine disorders of various grades depending on enzyme activity and exposure time were observed in patients with IMDs. To our knowledge, the present study is the first investigation that included mostly pediatric cases with IMDs.

In IMDs, a short stature usually results from multiple causes. These may include liver failure, renal failure, malnutrition, psychosocial causes or primary disease (2). There are a small number of hypopituitarism-induced growth retardation cases reported in the relevant literature, such as a few mitochondrial cytopathies (5) and iron-overload diseases (6,7,8). Growth retardation may occur in approximately 30%-60% of patients with mitochondrial cytopathy, cystinosis, and galactosemia (2). In our study, the overall proportion of growth retardation was 10.5%; however, this figure was 25% in patients with mitochondrial disease, and no growth retardation was observed in our seven patients with galactosemia (Table 1).

In this study, although no significant correlation between protein restriction and growth retardation was found, a serum albumin level below 3.5 g/dL was found to correlate with growth retardation. There is a consensus in the literature that an insufficient protein intake results in growth retardation (9). Hence, the main cause of growth retardation in cases with IMDs seems to be inadequate energy intake or strict protein restriction. To our knowledge, GH/IGF-1 axis in IMDs has not been evaluated so far. In our 27 cases with growth retardation, no abnormality was found concerning the GH/IGF-1 axis. An explanation for this finding might be that the growth retardation occurred independent of the GH/IGF-1 axis in our cases with IMDs. However, further studies should be conducted to find out the exact etiology of growth retardation in this group of the patients.

Many IMDs are accompanied by DM which usually occurs due to insulinopenia resulting from impaired pancreatic β-cell function (2). Among intoxication-type IMDs, DM develops due to iron overload in hemochromatosis (10) and in aceruloplasminemia (11), which may arise from ketoacidosis-induced pancreatitis in organic aciduria (12,13). In mitochondrial diseases (14,15) and glycogen storage diseases, DM is due to impaired β cell function arising from non-production of ATP (16). In addition, hypertriglyceridemia-induced pancreatitis may also contribute to the development of DM in glycogen storage diseases (17). In only 2 of 260 (0.07%) patients with IMDs, DM was diagnosed. One of these patients was diagnosed with LCAT deficiency of the lipid metabolism disorders, and another patient was diagnosed with KSS of the mitochondrial diseases. DM was previously described in both LCAT deficiency and KSS (2,18).

In adrenoleukodystrophy and in defects of energy metabolism of the IMDs, glucocorticoid deficiency may be observed (2). Among IMDs, X-linked adrenoleukodystrophy (X-ALD) is the disease that gives rise to primary adrenal insufficiency most frequently (19,20). Adrenal insufficiency occurs after the age of 3 years, which is likely to be the first indication of the disease. In Fabry disease, subclinical adrenal insufficiency may occur. Faggiano et al (4) found partial adrenal insufficiency in one of 18 patients with Fabry disease by using a corticotropin stimulation test. Mitochondrial disease-related adrenal insufficiency is rare in childhood, however, this may be the first symptom (21) associated with the poor prognosis (22,23,24). Glucocorticoid deficiency was detected in only 1 of 248 (0.4%) patients whose serum ACTH and cortisol levels were measured, and the diagnosis was glycerol kinase deficiency.

In conclusion, endocrine disorders of various grades depending on enzyme activity and exposure time can be observed in patients with IMDs. To our knowledge, this is the first study investigating endocrine functions in a group of patients with IMDs who were mostly of pediatric ages. In our study, an endocrine disorder was found only in 1.1% (3 of 260). GH/IGF-1 axis does not seem to be attributable to the growth retardation which was observed in 10.5% of the patients with IMDs. Serum albumin levels may be used in the follow-up of the patients to prevent growth retardation. We suggest that in pediatric patients with a diagnosis of IMD, routine endocrine tests are not necessary. Instead, both the patients and the IMD types should be evaluated individually, and endocrine tests should be done if required after obtaining a detailed history and performing physical examination including anthropometric and pubertal evaluation for the endocrine assessment.

## Ethics

Ethics Committee Approval: This study was conducted based on approval from the Ethical Committee of Medical Faculty of Uludağ University numbered 2013-18/32, as well as in accordance with the Declaration of Helsinki, Informed Consent: It was taken.

Peer-review: Externally peer-reviewed.

## Figures and Tables

**Table 1:**
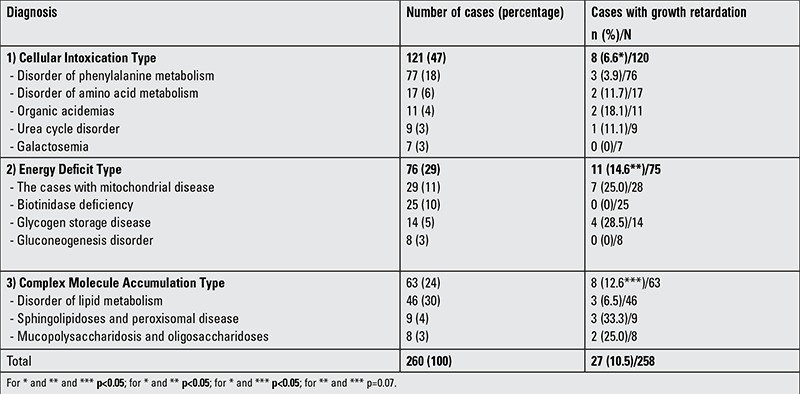
Numbers of patients in the main and sub-groups of inherited metabolic diseases and percentages of cases with growth retardation in the sub-groups of diseases
